# Mitoxantrone pleurodesis to palliate malignant pleural effusion secondary to ovarian cancer

**DOI:** 10.1186/1472-684X-3-4

**Published:** 2004-09-09

**Authors:** Nikolaos Barbetakis, Michalis Vassiliadis, Konstantinos Kaplanis, Rosalia Valeri, Christodoulos Tsilikas

**Affiliations:** 1 Cardiothoracic Surgery Department, Theagenio Cancer Hospital, A. Simeonidi 2, Thessaloniki, Greece; 2 Anaesthesiology Department, Theagenio Cancer Hospital, A. Simeonidi 2, Thessaloniki, Greece; 3 Gynaecologic Oncology Department, Theagenio Cancer Hospital, A. Simeonidi 2, Thessaloniki, Greece; 4 Cytopathology Department, Theagenio Cancer Hospital, A. Simeonidi 2, Thessaloniki, Greece

## Abstract

**Background:**

Advanced ovarian cancer is the leading non-breast gynaecologic cause of malignant pleural effusion. Aim of this study was to assess the efficacy of mitoxantrone sclerotherapy as a palliative treatment of malignant pleural effusions due to ovarian cancer.

**Methods:**

Sixty women with known ovarian cancer and malignant recurrent symptomatic pleural effusion were treated with chest tube drainage followed by intrapleural mitoxantrone sclerotherapy. Survival, complications and response to pleurodesis were recorded. The data are expressed as the mean ± SEM and the median.

**Results:**

The mean age of the entire group was 64 ± 11,24 years. The mean interval between diagnosis of ovarian cancer and presentation of the effusion was 10 ± 2,1 months. Eighteen patients (30%) had pleural effusion as the first evidence of recurrence. The mean volume of effusion drained was 1050 ± 105 ml and chest tube was removed within 4 days in 75% of patients. There were no deaths related to the procedure. Side effects of chemical pleurodesis included fever (37–38,5°C) chest pain, nausea and vomiting. At 30 days among 60 treated effusions, there was an 88% overall response rate, including 41 complete responses and 12 partial responses. At 60 days the overall response was 80% (38 complete responses and 10 partial responses). The mean survival of the entire population was 7,5 ± 1,2 months.

**Conclusions:**

Mitoxantrone is effective in the treatment of malignant pleural effusion secondary to ovarian cancer without causing significant local or systemic toxicity.

## Background

Cancer accounts for 40% of all pleural effusions, especially in patients over 50 years old [1]. Bronchogenic and breast cancer account for 75% of malignant pleural effusions, with the remaining 25% represented by a cross-section of other neoplastic diseases [2].

Approximately two thirds of malignant pleural effusions occur in women because of the strong association with breast and ovarian cancer [3]. Advanced ovarian cancer is the leading non-breast gynaecologic cause of malignant pleural effusion. Pleural metastases were found in 48% of women who died from ovarian cancer [4].

The general approach to managing malignant effusions is determined by symptoms, performance status of the patient, expected survival and response of the known primary tumor to systemic treatment. Intervention options range from observation in the case of asymptomatic effusions through simple thoracentesis to more invasive methods such as thoracoscopy, pleuroperitoneal shunting and pleurectomy. In patients with reasonable survival expectancy and good performance status every attempt should be made to prevent recurrence of the effusion. Intercostal tube drainage with instillation of a sclerosing agent, resulting in the obliteration of the pleural space, is the most widely used method to control recurrent symptomatic malignant pleural effusions.

Aim of this study was to study 60 patients with ovarian cancer who had a pleural effusion as a direct consequence of metastatic disease and to assess the efficacy of mitoxantrone as a sclerosing agent.

## Methods

Over an 8-year period (1996–2003), all patients with known ovarian malignancy and recurrent symptomatic malignant pleural effusion referred to Thoracic Surgery Department of Theagenio Cancer Hospital for drainage and sclerotherapy, were eligible to participate in this study. This study was approved by the Theagenio Cancer Research Ethics Committee and patients were included after giving their informed consent.

All patients satisfied the following eligibility criteria:

1. Known ovarian malignancy.

2. Recurrent symptomatic malignant pleural effusion. The diagnosis established by positive pleural fluid cytology on thoracentesis or positive pleural biopsy.

3. Evidence of expansion of the lung after fluid drainage and abscence of bronchial obstruction and/or fibrosis preventing lung expansion.

4. No previous intrapleural therapy.

5. Predicted survival of >1 month.

Patients were ineligible if they had a history of cardiac disease, obstructive jaundice or surgery within the previous month. No patient had systemic chemotherapy immediately prior to or during the first 30-day interval following sclerotherapy. Sixty women fulfilled the above eligibility criteria.

Pretreatment assessment was performed during admission and included history and physical examination, full blood count, liver biochemistry, electrocardiogram, a pre-drainage base line posteroanterior and lateral chest radiograph and other imaging as clinically indicated.

A chest tube (28–32 F) was inserted into the midaxillary line through the 5^th ^or 6^th ^intercostal space under local anesthesia and in some case additional intravenous benzodiazepines and/or narcotics. The pleural effusion was drained to dryness initially by gravity and followed if necessary by suction from a wall-mounted suction pump using a pressure of 20 cm H_2_O usually for 12–24 hours to achieve complete drainage of the effusion and lung re-expansion. Daily tube outputs were recorded and when drainage fell below 100 ml in a 24 h period, posteroanterior and lateral chest radiographs were obtained to assure that the fluid had been sufficiently evacuated, there were no loculated collections and the lung had fully re-expanded. Then the patients were eligible for pleurodesis.

Fifty ml of normal saline solution containing 2 mg/kg lidocaine were infused through the chest tube. After 15 minutes, a pleurodesis solution containing a mixture of 40 mg mitoxantrone and 20 ml normal saline was infused into the pleural cavity, after which the tube was clamped for 2 hours, while the patients changed position (rotated 90°) every 15 minutes. The tube then was re-opened. If the post-sclerotherapy drainage was <100 ml per day thetube was removed.

Complications related to the procedure were recorded. Post-sclerotherapy posteroanterior and lateral chest radiographs were obtained immediately after tube removal in order to be compared with others obtained 30 and 60 days later.

The radiographic response was determined on posteroanterior and lateral chest radiographs by observing the level of fluid meniscus overlying the costophrenic or vertebrophrenic angles and was determined as follows: complete response(CR) – no re-accumulation of pleural fluid, partial response (PR) – fluid recurrence less than 50% of the original level without symptoms or not requiring repeat drainage, progressive disease (PD) – re-accumulation to or above the original level with symptoms and requiring repeat drainage.

Survival was calculated from the day of diagnosis of pleural effusion to the day of death or to the last day of follow up if alive.

The data are expressed as the mean ± SEM and the median.

## Results

Sixty women were included in this study. The mean age of the entire group was 64 ± 11,24 years. The interval between diagnosis of ovarian cancer and the development of a subsequent malignant pleural effusion ranged from 1 to 36 months (mean: 10 ± 2,1 months). Fifty one patients (85%) had unilateral effusion and 9 (15%) bilateral. Histology, degree of differentiation and TNM stage [5] at the time of diagnosis of the primary tumor are shown in Table 1.

**Table 1:** Histology, degree of differentiation and TNM stage at the time of diagnosis of the primary tumor.

**Histology**	**Number of patients (n:60)**	Percentage
Serous cystadenocarcinoma	38	63,3%
Mucinous adenocarcinoma	8	13,4%
Mixed	4	6,7%
Clear cell	3	5%
Endometrioid	2	3,4%
Unknown	5	8,4%
**Degree of differentiation**		
G1	16	26,7%
G2	18	30%
G3	26	43,3%
**TNM stage**		
I	8	13,3%
II	18	30%
III	22	36,7%
IV	12	20%

Eigtheen patients (30%) had pleural effusion as the first manifestation of recurrent disease, whereas 42 patients (70%) were already diagnosed as having local or distant spread before the onset of pleural effusion. These 42 patients with preexisting metastases showed a variable pattern of secondary spread. Eighteen patients had parenchymal liver metastases and intraabdominal lymph nodes, 11 patients had liver metastases only, 8 had synchronous lung and liver metastases, 2 had lung metastases only, 1 patient had umbilical nodule, 1 had anterior wall abdominal wall infiltration and 1 had brain metastases.

The mean volume of effusion drained was 1050 ± 105 ml (range: 450–1500 ml). Chest tube was removed within 4 days in 75% of patients (range: 3 – 10 days).

There were no deaths related to the thoracostomy procedure. One patient experienced vasovagal reflex during the procedure with systemic hypotension and intense pleuritic pain. Hypotension was treated with intravenous fluids and the pain was controlled with narcotics. This episode lasted 20 minutes. The patient recovered without incident.

The most frequent complications related to pleurodesis were fever (temperature > 37°C), chest pain, nausea and vomiting (Table 2).

**Table 2:** Complications related to chemical pleurodesis with mitoxantrone

**Complications**	**Number of patients (n:60)**
None	31 (51,6%)
Fever	16 (26,6%)
Chest pain	12 (20%)
Nausea	11 (18,3%)
Vomiting	9 (15%)
Diarrhea	4 (6,6%)
Alopecia	1 (1,6%)
Skin Rash	1 (1,6%)
Dyspnea	1 (1,6%)
Myelosuppression	1 (1,6%)

Three patients died within 1 month of pleurodesis due to rapid progression metastatic disease. At 30 days, 57 patients were alive and 41 out of them had a complete response and 12 had a partial response. The overall response to chemical pleurodesis with mitoxantrone was 88% (53/57 patients). Four patients had progressive disease and revealed reaccumulation of fluid to or above the original level.

At 60 days 52 patients were alive and 38 out of them had a complete response and 10 had a partial response. The overall response was 80% (complete response 38/60 patients – 63,4%, partial response 10/60 patients – 16,6%). Follow up ranged from 10 days to 38 months with a mean of 10 ± 1,36 months. Eight patients out of the 41, who initially had complete response developed later recurrent pleural effusion and needed again tube thoracostomy and a second attempt of chemical pleurodesis.

The mean survival of the entire study population was 7,5 ± 1,2 months (median: 5,4 months).

## Discussion

Management of malignant pleural effusions depends on the underlying malignancy, extent of disease, potential effectiveness of treatment and performance status. In patients with lymphoma, small cell lung cancer or germ cell neoplasms, pleural effusions may be controlled initially by systemic therapy alone. In patients with metastatic breast or non small cell lung carcinoma, local palliative treatment is often required. Since malignant pleural effusions are frequently a preterminal event with a 30-day mortality rate of 29 to 50%, treatment is directed toward symptomatic relief with minimal discomfort, inconvenience and cost [6-8].

Local treatment options include repeated thoracenteses, chest tube drainage with sclerotherapy, pleuroperitoneal shunt or pleurectomy. Repeated thoracentesis is usually a temporizing measure and carries the risk for pneumothorax and pleural infection [9]. Inpatient drainage with large-bore tubes (28–36 F) is effective, with variable 30-day success rates reported between 55% and 95% [10]. For this reason, large-bore tube thoracostomy with sclerotherapy has become the most common palliative treatment for malignant effusions. It has to be mentioned that recent studies have shown that small drainage catheters (10 to 14 F) are as effective as large bore chest tubes in the treatment of malignant effusions [11]. Using imaging guidance, small tubes can be placed into loculated collections, are well tolerated and have complication rates less than the larger tubes [12].

Pleural effusion due to metastatic ovarian cancer is a frequent phenomenon and as shown in our study it can occur as early as one month or as late as 36 months with a median of 8,5 months. This is in complete accord with the study of Cheng et al who found a median interval of 9 months [13]. When effusion occurs within 1 month of diagnosis of ovarian cancer, one is probably dealing with IV stage and clinical experience has proved that these patients have an especially poor prognosis.

Numerous sclerosing agents have been used to treat malignant pleural effusions. Until recently, tetracycline was the most commonly used sclerosing agent with response rates ranging from 25 to 100% [14,15]. Because the intravenous form of tetracycline is no longer available, doxycycline has been proposed as an alternative.

Bleomycin has been studied extensively as a sclerosing agent [16,17]. Goff et al succesfully used bleomycin intrapleurally to treat malignant pleural effusions from gynecological cancer with a 71% overall response at 30 days and minimal adverse reactions. Intrapleural instillation is usually well tolerated but a few patients may report mild fever or transient nausea. Pleuritic pain and rigors are rarely reported side effects. This relative lack of systemic toxicity is likely due to limited absorption of bleomycin (approximately 40%) of the pleural cavity [18]. At 30 days bleomycin has been reported to be superior to tetracycline [19].

Talc has proved to be one of the most effective sclerosing agents for treating malignant pleural effusions. Talc causes severe pleuritis resulting in effective pleurodesis but can worsen dyspnea and can result in respiratory failure [20]. Other complications associated with talc pleurodesis include fever, acute pneumonitis, granulomatous pneumonitis and empyema [21]. Talc is instilled either as a slurry via chest tube or insufflated via thoracoscope.

Many other chemotherapeutic agents such as doxorubicin, cisplatin and cytarabine combination, etoposide, fluorouracil and mitomycin have been used for sclerotherapy. In addition radioactive isotopes, corynebacterium parvum, interferon and recombinant interleukin-2 have been instilled in the pleural space for treatment of malignant pleural disease. Response rate have been variable and less than optimal. Side effects are not inconsequential and thus none of these agents have gained widespread use [22].

Mitoxantrone is a synthetic anthracenedione which has been demonstrated to be effective in the treatment of peritoneal and pleural effusion. From a pharmacological point of view, mitoxantrone may be an especially appropriate choice due to its higher molecular weight and polarity since this may be factor important in prolonging contact with the pleura. The mechanism of intrapleural action of mitoxantrone has not yet been established. Both the inflammatory and antineoplastic activity of mitoxantrone intrapleurally have been described [23,24].

Our findings are consistent with the findings of others. In a prospective study in 18 patients, Musch et al [25] reported a 30-day success rate of 75%. A comparative study including bleomycin and mitoxantrone showed almost an equal 30-day response of 64% and 67% respectively [26]. Van Belle et al [27] had an overall 30-day response of successful pleurodesis of 67% in patients with ovarian cancer (2/3 patients). Morales et al [28] treated a group of 21 patients with malignant pleural effusions, with instillation of mitoxantrone with a 100% response and no toxic effects.

There is only one study which proved mitoxantrone ineffective. Groth et al. [29] presented a prospective randomized trial on the treatment of malignant pleural effusions with intrapleural mitoxantrone versus placebo (pleural tube alone with instillation of isotonic NaCl). Their data suggest no statistically significant difference between the two arms with respect to response and response duration.

Our study confirmed the majority of previous reports that mitoxantrone is an effective agent in controlling recurrent malignant pleural effusions. The overall 30-day response rate was 88%. Side effects were mild and rare.

To develop new treatment plans for the management of pleural effusions, one must consider several requirements. First, no treatment regimen should exacerbate patients' symptoms, since palliation is the main aim. Second, seriously ill patients should not be subjected to procedures associated with high mortality and morbidity. Third, since about half the patients with pleural effusion will have no other clinically apparent metastases, treatment should be local rather than systemic. To be successful, the local treatment has to be effective and given at the first sign of the effusion, because inadequate or delayed treatment may eliminate the possibility of any subsequent therapy being effective, by producing loculation of the effusion.

## Conclusions

Pleural effusion often occurs during the course of ovarian cancer. Chemical pleurodesis via bedside thoracostomy has been shown to be effective and has become a common therapeutic approach. Using this approach we found mitoxantrone to be highly effective at controlling malignant pleural effusions and decreasing the associated symptoms of dyspnea and pain. Our data justify further studies in a controlled setting to elucidate the biological action and prognostic relevance of mitoxantrone in the treatment of malignant pleural effusions and to compare this agent with other treatment procedures.

## Competing interests

None declared.

## Authors's contributions

NB conceived the study.and performed thestatistical analysis. MV and RV participated in the design of the study. KK and CT conceived of the study and participated in its coordination. All authors read and approved the final manuscript.

## Pre-publication history

The pre-publication history for this paper can be accessed here:


